# Differential direct effects of cyclo-oxygenase-1/2 inhibition on proteoglycan turnover of human osteoarthritic cartilage: an *in vitro *study

**DOI:** 10.1186/ar1846

**Published:** 2005-11-09

**Authors:** Simon C Mastbergen, Nathalie WD Jansen, Johannes WJ Bijlsma, Floris PJG Lafeber

**Affiliations:** 1 Rheumatology & Clinical Immunology, University Medical Center Utrecht, Utrecht, The Netherlands

## Abstract

Treatment of osteoarthritis (OA) with nonsteroidal anti-inflammatory drugs (NSAIDs) diminishes inflammation along with mediators of cartilage destruction. However, NSAIDs may exert adverse direct effects on cartilage, particularly if treatment is prolonged. We therefore compared the direct effects of indomethacin, naproxen, aceclofenac and celecoxib on matrix turnover in human OA cartilage tissue. Human clinically defined OA cartilage from five different donors was exposed for 7 days in culture to indomethacin, naproxen, aceclofenac and celecoxib – agents chosen based on their cyclo-oxygenase (COX)-2 selectivity. As a control, SC-560 (a selective COX-1 inhibitor) was used. Changes in cartilage proteoglycan turnover and prostaglandin E_2 _production were determined. OA cartilage exhibited characteristic proteoglycan turnover. Indomethacin further inhibited proteoglycan synthesis; no significant effect of indomethacin on proteoglycan release was found, and proteoglycan content tended to decrease. Naproxen treatment was not associated with changes in any parameter. In contrast, aceclofenac and, prominently, celecoxib had beneficial effects on OA cartilage. Both were associated with increased proteoglycan synthesis and normalized release. Importantly, both NSAIDs improved proteoglycan content. Inhibition of prostaglandin E_2 _production indirectly showed that all NSAIDs inhibited COX, with the more COX-2 specific agents having more pronounced effects. Selective COX-1 inhibition resulted in adverse effects on all parameters, and prostaglandin E_2 _production was only mildly inhibited. NSAIDs with low COX-2/COX-1 selectivity exhibit adverse direct effects on OA cartilage, whereas high COX-2/COX-1 selective NSAIDs did not show such effects and might even have cartilage reparative properties.

## Introduction

Nonsteroidal anti-inflammatory drugs (NSAIDs) are widely used to alleviate the symptoms of osteoarthritis (OA) [1]. OA is a slowly progressive degenerative joint disease, with a high incidence [2], and is characterized by gradual loss of articular cartilage [3]. Clinical efficacy and side effects in terms of gastrointestinal problems are mostly well understood [4], although cardiovascular side effects of second-generation NSAIDs, namely the selective COX-2 inhibitors, only recently became evident [5,6]. However, such adverse effects have always been a concern for conventional NSAIDs [7]. In addition, the side effects of NSAIDs and selective COX-2 inhibitors on articular (osteoarthritic) cartilage tissue are controversial.

Direct effects of NSAIDs on cartilage may be important, particularly in the treatment of joint disease in which inflammation is only mild and secondary (as in OA) and when treatment is chronic. Thus, although NSAIDs may be useful in reducing pain and inflammation in OA, if they have adverse direct effects then they may enhance the process of cartilage degeneration by interfering with intrinsic repair activities. If NSAIDs do have such direct adverse effects then these should be considered in addition to the gastrointestinal and cardiovascular effects when one is prescribing NSAIDs for management of OA.

*In vitro *studies have shown that several types of conventional NSAIDs (such as sodium salicylate and indomethacin) inhibited the synthesis of cartilage matrix components, whereas others (such as aceclofenac and meloxicam) increased matrix synthesis and protected the chondrocytes against apoptosis [8-12]. Other NSAIDs (for instance piroxicam) had no effect. Studies in animal models of OA verified that NSAIDs had detrimental or favourable actions on OA progression [13-16], although the same NSAIDs had diverse effects on articular cartilage in different studies, depending on the animal model used [15,16].

With respect to the second-generation NSAIDs fewer data are available. We recently showed a beneficial effect of celecoxib (Celebrex; Pfizer Inc., New York, NY, USA) on normal cartilage under the influence of interleukin-1 and tumour necrosis factor-α; in normal healthy cartilage no effects were observed [17]. Findings reported by El Hajjaji and coworkers [18] showed that celecoxib was able to increase proteoglycan synthesis and to diminish proteoglycan release in OA cartilage obtained at joint replacement surgery. Recent findings reported by our group [19] confirmed these data and demonstrated that celecoxib had a favourable effect on proteoglycan synthesis, retention, release and content in both degenerated (preclinical) and (late-stage) OA cartilage.

NSAIDs inhibit both COX-1 and COX-2 [20]. This inhibition appears to be correlated with the well characterized gastrointestinal toxicity, with those agents with more COX-1 selectivity having a tendency to cause more gastrointestinal damage [21]. In contrast, a more recent debate centers on whether the more COX-2 selective agents carry greater risk for cardiovascular side effects [5,6]. In the present study we considered whether the direct effects of NSAIDs on cartilage are dependent on their COX-2 selectivity or lack thereof. It could well be that the adverse effects on cartilage of some of the conventional NSAIDs result from inhibition of COX-1.

For this reason, the present study was conducted to evaluate the *in vitro *effects of several frequently used NSAIDs on human OA articular cartilage. Effects of indomethacin and naproxen (nonselective NSAIDs with moderate COX-1 selectivity) [20] were compared with those of aceclofenac (moderately selective for COX-2 [21]) and the selective COX-2 inhibitor celecoxib, covering a range from COX-1 to COX-2 selectivity.

## Materials and methods

### Cartilage culture technique

OA cartilage obtained from patients at knee replacement surgery with diagnosed OA was obtained postoperatively. NSAID medication was stopped 7 days before surgery, so ensuring that there would be no confounding effect of previous medication. Cartilage that appeared to be full thickness with significant fibrillation was selected [22], so the entire joint had a worse appearance than represented by the cartilage used in the evaluation. Cartilage bone samples were stored in phosphate-buffered saline for no longer than 4 hours. Collection of cartilage was done according to the medical ethical regulations of the University Medical Centre Utrecht.

Slices of cartilage were cut aseptically as thick as possible from the articular bone surface (excluding the underlying bone), cut into square pieces, weighed aseptically (range 5–15 mg [accuracy ± 0.1 mg]) and cultured individually in 96-well round-bottomed microtitre plates (200 μl culture medium, 5% carbon dioxide in air, 37°C). The culture medium consisted of Dulbecco's modified Eagle's medium, supplemented with glutamine (2 mmol/l), penicillin (100 IU/ml), streptomycin sulphate (100 μg/ml), ascorbic acid (0.085 mmol/l) and 10% heat inactivated pooled human male AB^+ ^serum. Cartilage was always precultured for 24 hours (washout period), after which culture medium was refreshed before the start of the experiment.

In addition, three tissue samples from each donor were fixed in 4% phosphate-buffered formalin for standard light microscopy. Sections were stained with safranin-O fast green-iron haematoxylin and graded for features of OA according to the slightly modified criteria [23] presented by Mankin and coworkers [24]. The tidemark between cartilage and bone was not present in our cartilage samples and cartilage samples were not covered with pannus. Therefore, the maximum score that could be obtained was 11, rather than the original 14 if all criteria described by Mankin and coworkers [24] (including pannus, clefts to calcified zone and tidemark crossed by blood vessels) had been included.

### Experimental setup

OA human articular cartilage tissue was cultured for 7 days in the absence or presence of the following additives: indomethacin (10 μmol/l; Sigma, St. Louis, MO, USA), naproxen (300 μmol/l; Sigma, St. Louis, MO, USA), aceclofenac (0.03 μmol/l; UCB Pharma, Chemin du Foriest, Belgium), or celecoxib (1 μmol/l; supplied by Pfizer Inc., New York, NY, USA). Final concentrations resembled the mean pharmacological plasma concentrations of each of the NSAIDs [25-27]. In addition, SC-560 (0.1 μmol/l; Sigma) – an experimental COX-1 inhibitor – was added. A concentration of 0.1 μmol/l guarantees COX-1 selectivity; higher concentrations also inhibit COX-2. After 4 days the medium was refreshed and cartilage cultured for a successive 3 days with the same additives. Changes in cartilage matrix turnover (proteoglycan synthesis, retention and release) and matrix integrity (proteoglycan content) were determined. Experiments were repeated five times, using cartilage from a different donor in each case.

### Proteoglycan analyses

Sulphate incorporation rate – a measure of the rate of proteoglycan synthesis – was determined during the last 4 hours of the first 4-day culture period, as described previously [28]. Before addition of ^35^SO_4_^2- ^(Na_2 _^35^SO_4_, 14.8 kBq/200 μl, DuPont NEX-041-H, carrier free), culture medium was replaced by equilibrated (carbon dioxide and temperature) fresh medium. After 4 hours of labelling, the cartilage explants were rinsed three times for 45 minutes in culture medium under culture conditions and incubated for an additional 3 days. After this second culture period medium was removed and the samples were stored at -20°C for further analysis. Cartilage tissue samples were digested (2 hours, 65°C) in papain buffer, as described previously [22]. Papain digests were diluted to the appropriate concentrations and glycosaminoglycans (GAGs) were stained and precipitated with Alcian Blue dye solution [29]. The pellet obtained after centrifugation (9,000 *g*, 10 minutes) was washed once (NaAc-buffer containing 0.1 mol/l MgCl_2_) and subsequently dissolved in SDS. The ^35^SO_4_^2- ^radioactivity of the samples was measured by liquid scintillation analysis. ^35^SO_4_^2- ^incorporation was normalized to the specific activity of the medium, labelling time and wet weight of the cartilage samples. Proteoglycan synthesis rate is expressed as percentage change compared with untreated control values.

Release of newly formed proteoglycans as a measure of retention of these proteoglycans was similarly determined. GAGs were precipitated from the medium obtained from days 4–7 with Alcian Blue [29]. The radiolabelled GAGs were measured by liquid scintillation analysis and normalized to the proteoglycan synthesis rate. Percentage release of newly formed proteoglycans is expressed as percentage change compared with untreated control values.

For the total release of proteoglycans, the GAG in the medium obtained from days 4–7 were precipitated and stained with Alcian Blue as described above. The GAG content in the papain digest of cartilage samples, as a measure of proteoglycan content, was analyzed in the same way. Blue staining was quantified photometrically by the change in absorbance at 620 nm. Chondroitin sulphate (Sigma C4383) was used as a reference. Values for content were normalized to the wet weight of the cartilage and expressed as percentage change compared with untreated control values. Values for release were normalized to the GAG content of the explants. Percentage release of GAGs is expressed as percentage change compared with untreated control values.

### Prostaglandin E_2 _determination

Prostaglandin (PG)E_2 _was determined in culture medium at day 4 by enzyme immunoAssay (Caymann Chemical, Ann Arbor, MI, USA) and expressed as percentage change compared with control.

### Calculations and statistical analysis

Because of focal differences in composition and bioactivity of the cartilage in the knee joint, the results of 10 cartilage samples per parameter per donor, obtained randomly and handled individually, were averaged and taken as a representative value for the cartilage of that donor. Several experiments with each cartilage sample from the different donors (*n *= 5) were performed. Statistical evaluation of the effects of a single intervention (for example NSAIDs) compared with untreated cartilage from the same donors was performed with a nonparametric test for paired data (Wilcoxon). For statistical evaluation of differences between different interventions, the percentage change compared with untreated cartilage from the same donors was calculated. The effects of different treatments were compared using a nonparametric test for unpaired data (Mann-Whitney). *P *≤ 0.05 were considered statistically significant.

## Results

### Effects of selective versus nonselective NSAIDs on osteoarthritic cartilage

**Figure 1:**
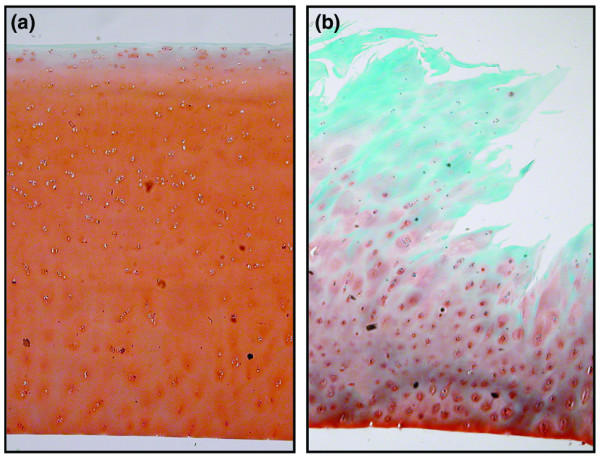
Normal healthy and osteoarthritic cartilage histology. Representive light micrographs of condylar cartilage obtained *post mortem *from joints with **(a)** normal healthy cartilage and **(b)** cartilage obtained at joint replacement surgery. Sections are stained with safranin-O fast green-iron haematoxylin and graded for features of osteoarthritis according to the slightly modified criteria [23] described by Mankin and coworkers [24]; scores for the shown samples are 0 and 7, respectively.

OA cartilage from the different donors had on average a modified Mankin score of 5 ± 1. It should be kept in mind that only the cartilage that could be cut from the joint surfaces after replacement surgery was used. Thus, the entire joint had a worse appearance than that indicated by the modified Mankin score of the cartilage used. Surface deterioration of the OA cartilage was clearly visible by light microscopy. An example of a severely affected cartilage tissue explant is shown in Figure 1b; this contrasts with the normal healthy cartilage shown in Figure 1a. The latter was obtained from a healthy joint (*post mortem*) that was not used in the present study. The safranin O staining was lost from the surface layer of the OA samples, and chondrocyte distribution was disturbed (clusters of chondrocytes in the surface layer of the cartilage were visible; Figure 1b).

The OA cartilage exhibited typical basal biochemical features in terms of proteoglycan turnover (Table 1): low proteoglycan synthesis, high proteoglycan release (both newly formed proteoglycans and resident proteoglycans) and diminished proteoglycan content. Baseline data between the four groups did not differ significantly. Data obtained from healthy cartilage (*n *= 5; donor age 68 ± 5 years) from femoral condyles, given as a point of reference, are as follows: histological grade 0.7 ± 0.1; proteoglycan synthesis rate 12.5 ± 1.1 nmol/hour per g; percentage new proteoglycan release 7.1 ± 0.5%; total proteoglycan release 3.9 ± 0.5%; and proteoglycan content 29.2 ± 3.4 mg/g. This cartilage was obtained *post mortem *from donors without any history of joint disorders and was treated the same way over a similar time period.

**Figure 2:**
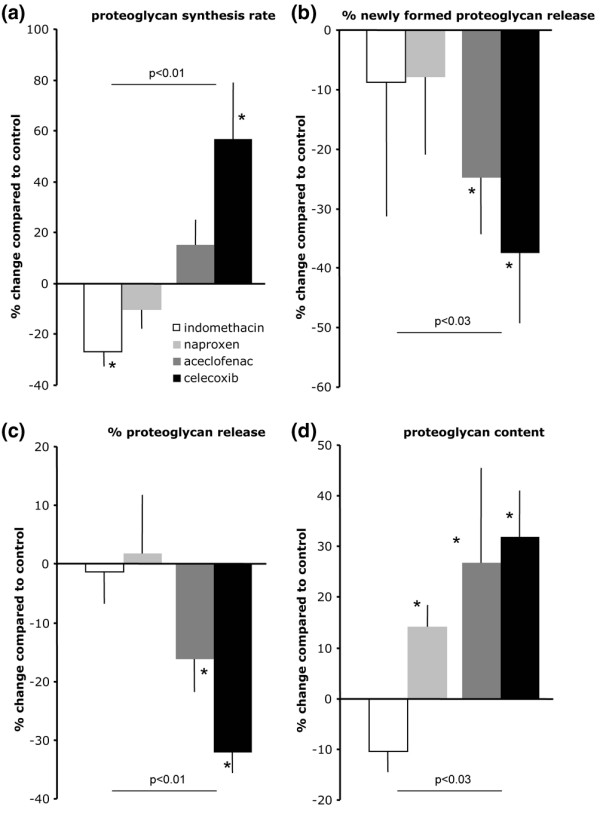
Effects of four NSAIDs on proteoglycan turnover and content of OA cartilage. Shown are the following measures of proteoglycan turnover: **(a) **percentage change in proteoglycan synthesis rate (compared with untreated osteoarthritic cartilage of the same donor), as a measure of cartilage matrix synthesis (proteoglycan synthesis); **(b) **percentage release of newly formed proteoglycans (new proteoglycan release), as a measure of retention of newly formed proteoglycans (normalized to the synthesis of these proteoglycans); **(c) **percentage total release of proteoglycans (total proteoglycan release), measured as the percentage release of glycosaminoglycans (normalized to the glycosaminoglycan content); and **(d) **proteoglycan content (proteoglycan content). White bars represent effects of indomethacin (10 μmol/l); light grey bars represent naproxen (300 μmol/l), dark grey bars represent aceclofenac (0.03 μmol/l) and black bars represent celecoxib (1 μmol/l). The results are presented as means of five experiments (with each cartilage sample from the different donors) ± standard error. Statistically significant differences for the effect of an NSAID compared with OA cartilage of the same donors is calculated using nonparametric paired analysis (*P < 0.05). Difference between percentage changes for the two nonselective NSAIDs compared with the (more) selective NSAIDs (*n *= 10 versus *n *= 10) is calculated by nonparametric, nonpaired analysis (*P *values are given for each of the parameters). NSAID, nonsteroidal anti-inflammatory drug; OA, osteoarthritis.

Indomethacin decreased proteoglycan synthesis in OA cartilage (-27 ± 6% compared with untreated control OA cartilage; *P *< 0.05; Figure 2, white bars). No significant effect was found on proteoglycan release, both newly formed and resident proteoglycans. There was a tendency toward a decrease in proteoglycan content (on average -11 ± 4%). Naproxen had no significant effects on proteoglycan turnover in OA cartilage, although there was a tendency for this agent to exert effects similar to those of indomethacin. Remarkably, naproxen resulted in a slight but statistically significant increase in proteoglycan content (14 ± 4%; *P *< 0.05; Figure 2, light grey bars).

In contrast to indomethacin and naproxen, treatment with aceclofenac, which is more selective for COX-2 inhibition [21], was associated with improvements in all parameters (Figure 2a, dark grey bars). Proteoglycan synthesis was on average increased by 15 ± 10%, although this finding was not statistically significant. However, this increased synthesis was combined with an improved retention of these newly formed proteoglycans, as reflected by the diminished rate of release of newly formed proteoglycans (-25 ± 10%; *P *< 0.05). This was also the case for the total proteoglycan release, which was reduced (-16 ± 6%; *P *< 0.05). More importantly, aceclofenac improved the proteoglycan content on average by 27 ± 19% (*P *< 0.05).

The most selective COX-2 inhibitor of the four tested, namely celecoxib, caused even greater improvement in proteoglycan parameters as compared with untreated controls (Figure 2, black bars). Proteoglycan synthesis increased 57 ± 22% (*P *< 0.05), whereas the release of those newly formed proteoglycans was reduced by 38 ± 12% (*P *< 0.05). A comparable reduction was found for total proteoglycan release (-32 ± 4%; *P *< 0.05). With respect to matrix integrity, celecoxib was able to improve the proteoglycan content by 32 ± 9 % (*P *< 0.05).

**Figure 3:**
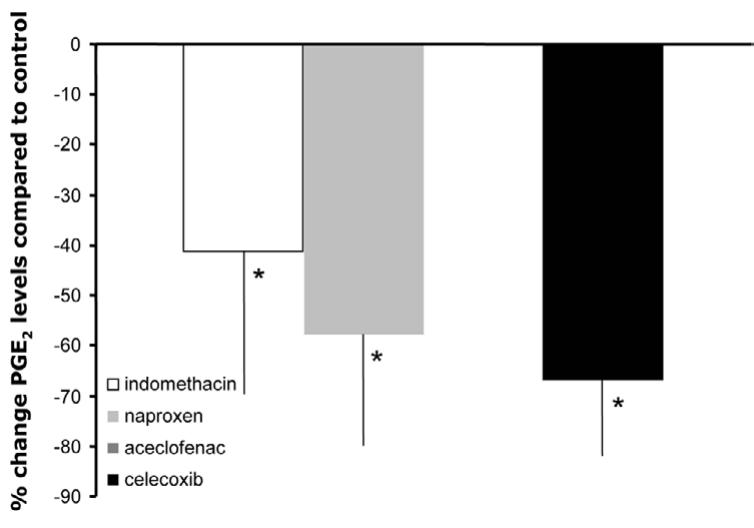
Effects of three of the four NSAIDs on PGE_2 _production in OA cartilage. Percentage changes in PGE_2 _levels in culture supernatants of osteoarthritic cartilage treated with different NSAIDs are shown. Mean values (*n *= 5 ± standard error) are presented for indomethacin (open bar), naproxen (light grey bar) and celecoxib (black bar). Effects of aceclofenac were not measured. Statistical differences of the effects of the different NSAIDs compared with untreated controls were calculated using nonparametric paired analysis (*P < 0.05). NSAID, nonsteroidal anti-inflammatory drug; OA, osteoarthritis; PG, prostaglandin.

When the effects of aceclofenac and celecoxib were compared with those of indomethacin and naproxen, the beneficial effects of the former were significantly different from the adverse effects of the latter in terms of proteoglycan synthesis, retention, release and content (Figure 2). All NSAIDs inhibited PGE_2_, as an indirect measure of COX inhibiting activity (on average, more than 60% inhibition for all compounds compared with untreated controls; *P *< 0.05; Figure 3). However, there was a tendency for greater COX-2 selectivity in an NSAID to correlate with more pronounced inhibition of PGE_2_. Unfortunately, the culture media from the aceclofenac samples could not be analyzed.

### Effects of SC-560 on osteoarthritic cartilage

Because it appeared that absence of selectivity for COX-2 inhibition resulted in no or even adverse direct effects on cartilage, we studied the effect of an experimental selective COX-1 inhibitor as well. In these experiments the average age of donors was 73 ± 3 years and they were all female. The average modified Mankin grade of these donors was 6 ± 1. The donors did not differ significantly from the other OA donors for any of the parameters given in Table 1.

**Figure 4:**
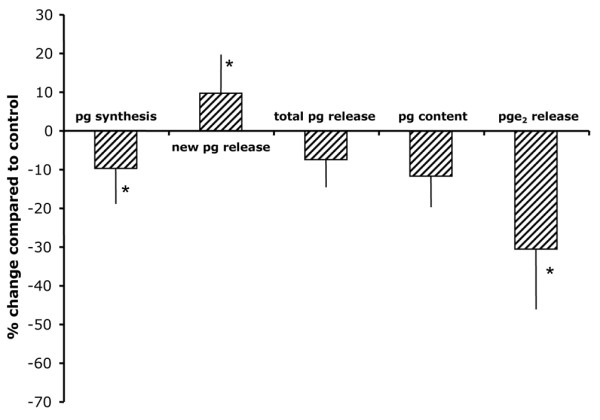
Effect of a selective COX-1 inhibition on OA cartilage. Shown are the percentage changes compared with healthy cartilage of proteoglycan synthesis rate as a measure of cartilage matrix synthesis (pg synthesis); percentage release of newly formed proteoglycans (new pg release) as a measure of retention of the newly formed proteoglycans (normalized to the synthesis of these proteoglycans); percentages total release of proteoglycans (total pg release), measured as the percentage release of glycosaminoglycans (normalized to glycosaminoglycan content); proteoglycan content (pg content) and prostaglandin E_2 _release (pge_2 _release) under the influence of 0.1 μmol/l SC-560 (a selective COX-1 inhibitor). The results are presented as means of five experiments (with each cartilage sample from the different donors) ± standard error. The absolute values of untreated controls are as follows: prostaglandin synthesis rate 3.4 ± 1.3 nmol/hour per g; % new prostaglandin release 11.4 ± 0.6%; total prostaglandin release 6.4 ± 1.1%; and prostaglandin content 17.9 ± 0.8 mg/g. Significant differences between SC-560 treated and untreated cartilage were calculated using nonparametric paired analysis (*P < 0.05).

When SC-560 was added to the OA cartilage cultures, an inhibition of proteoglycan synthesis (-10 ± 9% compared with untreated control; *P *< 0.05; Figure 4) was found. This was accompanied by enhanced release of newly formed proteoglycans (10 ± 10% compared with untreated control; *P *< 0.05). There was no statistically significant change in total proteoglycan release. The inhibition in synthesis and retention did not lead to a further statistically significant reduction in proteoglycan content, although there was a tendency toward a decrease in proteoglycan content. Inhibition of PGE_2 _production was relatively mild compared with that induced by NSAIDs (on average by 30 ± 15%; *P *< 0.05). The effects of this selective COX-1 inhibitor were not significantly different from those of the nonselective NSAIDs, but for all parameters they were statistically different from the (more) COX-2 selective NSAIDs (data not shown).

## Discussion

The purpose of this study was to evaluate the effect of frequently used NSAIDs on human OA articular cartilage *in vitro*. There was an emphasis on possible differences between conventional nonselective COX-2 inhibitors and the more selective COX-2 inhibitors, as recently classified by Warner and Mitchell [20]. It appeared that COX-2 selectivity resulted in cartilage reparative properties, whereas the absence of COX-2 selectivity could even result in negative effects. The adverse effects of an experimental COX-1 selective compound corroborate the latter finding.

Although we calculated that the concentrations of NSAIDs used *in vitro *were likely to be close to the concentrations of NSAIDs found *in vivo*, it should be considered that the actual concentrations reached *in vivo *in patients might be slightly different from the *in vitro *concentrations in this study because of low concentrations of binding proteins such as albumin (the culture medium included only 10% human serum) [30]. However, we expect the binding of the different NSAIDs to be comparable, and so the observed differences between the effects of the different NSAIDs should be consistent *in vitro *and *in vivo*.

The direct negative effects of indomethacin are mainly reflected by an inhibition of proteoglycan synthesis and diminished retention of these newly formed proteoglycans. This is in accordance with previous reports that examined frequently used NSAIDs. Indomethacin, naproxen and ibuprofen, tested under comparable *in vitro *conditions, are known to inhibit the synthesis of cartilage proteoglycans [9,10,31,32] and to increase the release of proteoglycans [9,10]. In addition, indomethacin was found to have deleterious effects on articular cartilage of both left and right knees in OA rats induced by injections of sodium iodoacetate in the right knee [9]. Indomethacin has also been demonstrated to affect glycosyltransferase; this is important for the synthesis of the polysaccharide chains of proteoglycans [33] and might have affected the measured sulphate incorporation rate. Also, naproxen exhibited adverse effects in an *in vivo *study using the ACLT canine model of OA [16]. That study found that naproxen increased water content in cartilage. However, a different ACLT canine study [15] showed that naproxen was able to suppress significantly the decreases in proteoglycan content and metalloproteinase activities in knee articular cartilage [15]. In our hands naproxen did not have a pronounced adverse effect on OA cartilage *in vitro*, as previously demonstrated, although there was a tendency toward such an effect. With respect to the direct effects of selective COX-2 inhibitors on cartilage, recent data showed a beneficial effect of celecoxib in normal cartilage under inflammatory conditions; normal healthy cartilage remained unaffected [17]. For OA cartilage obtained at joint replacement surgery, it was demonstrated that celecoxib could increase proteoglycan synthesis and diminish proteoglycan release [18]. Recently, our group confirmed these findings and showed that celecoxib had a favourable effect on proteoglycan synthesis, retention, release and content in both degenerated (preclinical) and (late-stage) OA cartilage [19]. Remarkably, the effects of aceclofenac, a derivate of diclofenac, were similar to those of celecoxib, suggesting a similar mechanism of action. Based on the classification presented by Warner and coworkers [21] diclofenac has a preference for COX-2; from this and our findings, we assume that aceclofenac has comparable selectivity. The metabolism of aceclofenac differs from that of diclofenac and is human specific [34]. The main metabolite of aceclofenac is 4-hydroxy-aceclofenac. The other metabolites, namely diclofenac and 4-hydroxy-diclofenac, account for only 5% of the administered dose [25]. Aceclofenac acts as a functional inhibitor of PGE_2 _production, either by acting directly on the production of cytokines that induce COX in the inflamed tissues [35] or by its preferential intracellular conversion to COX(-2) active metabolites [36,37], or most likely by both processes at the same time [37].

The experimental selective COX-1 inhibitor SC-560 had effects similar to those of indomethacin, indicating that inhibition of COX-1 results in an adverse effect on proteoglycan synthesis and retention. In contrast, when COX-2 is selectively inhibited in OA cartilage we found a beneficial effect with respect to proteoglycan turnover. These findings imply an important role for COX-2 in the disturbed proteoglycan turnover in OA, whereas COX-1 plays a more physiological role in the chondrocytes. This is in accordance with the generally held belief that COX-1 is the 'housekeeping' isoform of COX and has clear physiological functions. For instance, its activation leads to the production of prostacyclin, which when released by the endothelium is antithrombogenic and when released by the gastric mucosa is cytoprotective [38]. In contrast, COX-2 is excessively induced under inflammatory and detrimental conditions such as OA. This established concept has been modified by recent investigations demonstrating a significant participation of prostaglandins derived via the COX-1 pathway in some inflammatory processes [39-41], especially pain. Also, the recent identification of cardiovascular side effects of selective COX-2 inhibitors, and NSAIDs in general, forces us to reconsider the current concept. Nevertheless, in the case of proteoglycan turnover in OA cartilage the concept apparently still holds true.

COX-2 is expressed in OA tissues. The expression of COX-2 and PGE_2 _in OA meniscus, synovial membrane, osteophytic fibrocartilage and in the articular OA cartilage has been described [42]. However, when we selectively inhibited COX-1, thereby inhibiting only a relatively small amount of PGE_2_, proteoglycan turnover became worse (especially proteoglycan synthesis). This indicates that COX-1 inhibition results in alterations to products formed by COX-1 in a mechanism that is independent of PGE_2_, which influences proteoglycan turnover negatively. The difference in outcome when COX-1 or COX-2 is inhibited can be explained by the possibility that the two COX isoforms may actually have different primary products through preferential interaction with different terminal synthases [43], irrespective of the final product, namely PGE_2_. Another factor might be the different intracellular localization of the two COX isoforms, which might lead to (unknown) alternative effects of the same prostaglandin products [43]. Nevertheless, the upregulation of PGE_2 _in OA cartilage, together with the beneficial effects of COX-2 inhibition, implies an important role for COX-2 in OA cartilage and supports the use of selective COX-2 inhibitors in treatment of OA.

Other, COX independent effects of NSAIDs might be involved as well, however [44]. Several studies have demonstrated that certain NSAIDs, such as ibuprofen, cause anti-inflammatory effects independent of COX activity and prostaglandin synthesis inhibition [45-47]. These effects are mediated through inhibition of certain transcription factors such as nuclear factor (NF)-κB and activator protein-1 [48-50]. The respective NSAIDs might interfere directly with the transcription factors, but their effects are probably mediated predominantly through alterations of the activity of cellular kinases such as IKKβ, Erk, p38, or mitogen-activated protein kinase [51]. These effects are not shared by all NSAIDs, because indomethacin failed to inhibit NF-κB and activator protein-1 activation, as well as Erk activity [49,52,53]. In contrast, indomethacin is able to activate peroxisome proliferator-activated receptor-γ, which is not sensitive to sodium salicylate or aspirin [54]. At the concentration tested, celecoxib inhibits NF-κB, an effect also observed for other NSAIDs but only at higher concentrations [55]. Inhibition of NF-κB is related to inhibition of matrix metalloproteinases and aggrecanases [56]. These effects may add to the observed differences in direct effects of NSAIDs on cartilage.

Importantly, we discussed solely the direct effects of NSAIDs on cartilage. These effects should be seen within the context of the significant anti-inflammatory effects of these NSAIDs. By inhibiting joint inflammation, they may indirectly be beneficial to cartilage, specifically when inflammation is primary in the cause of cartilage damage, as is the case for rheumatoid arthritis. However, in OA, in which inflammation may contribute to but is not primarily responsible for cartilage damage, adverse direct effects of NSAIDs on cartilage with long-term treatment may have an important impact on long-term outcome. Therefore it remains important to extend these *in vitro *studies with animal *in vivo *studies and even clinical setups.

## Conclusion

Although they are *in vitro *findings, the results of the present study suggest that, in addition to the anti-inflammatory and analgesic characteristics of selective COX-2 inhibitors, their gastrointestinal and their cardiovascular side effects, the direct (side) effects of these NSAIDs on cartilage should also be considered in the choice of NSAID during chronic treatment of joint diseases such as OA.

## Abbreviations

COX = cyclo-oxygenase; GAG = glycosaminoglycan; NF-κB = nuclear factor-κB; NSAID = nonsteroidal anti-inflammatory drug; OA = osteoarthritis; PG = prostaglandin;

## Competing interests

This investigation was supported by an unrestricted grant from Pfizer and UCB Pharma.

## Authors' contributions

SM, JB, and FL conceived the study, participated in its design and coordination, and helped to draft the manuscript. SM and NJ carried out the experiments and performed all of the assays. All authors read and approved the final manuscript.

## Figures and Tables

**Table 1:** Histological and biochemical characteristics of baseline values of human osteoarthritic cartilage for different treatment groups

Parameter	Indomethacin (10 μmol/l)	Naproxen (300 μmol/l)	Aceclofenac (0.03 μmol/l)	Celecoxib (0.01 μmol/l)
Age (years)	70 ± 2	67 ± 3	63 ± 2	70 ± 3
Sex (female/male)	4/1	3/2	4/1	4/1
Histological cartilage damage (Mankin grade)	3 ± 1	4 ± 1	6 ± 2	5 ± 2
Prostaglandin synthesis rate (nmol/hour per g)	6.8 ± 2.3	3.4 ± 1.9	4.8 ± 0.9	5.5 ± 1.8
% New prostaglandin release	11 ± 2	13 ± 2	12 ± 2	11 ± 2
% Total release	9 ± 2	7 ± 2	5 ± 1	6 ± 1
Prostaglandin content (mg/g)	22 ± 2	19 ± 1	17 ± 6	19 ± 1
